# Longitudinal transitions in e-cigarette and cigarette use among US adults: prospective cohort study

**DOI:** 10.1016/j.lana.2023.100508

**Published:** 2023-05-16

**Authors:** Mohammad Ebrahimi Kalan, Noel T. Brewer

**Affiliations:** a School of Health Professions, Eastern Virginia Medical School, Norfolk, VA, United States; b Department of Health Behavior, Gillings School of Global Public Health, University of North Carolina, Chapel Hill, NC, United States; c Lineberger Comprehensive Cancer Center, University of North Carolina, Chapel Hill, NC, United States

**Keywords:** Electronic nicotine delivery systems (ENDS), E-cigarettes, Cigarettes, Behavioral transitions, PATH

## Abstract

**Background:**

To support tobacco control efforts, this study sought to characterize longitudinal transitions in use of electronic nicotine delivery systems (ENDS) and cigarettes.

**Methods:**

Participants were nationally representative samples of 53,729 US adults from Waves 3–5 (2015–2019) of the Population Assessment of Tobacco and Health Study. We examined behavioral transitions (initiation, relapse, progression, and cessation) in ENDS and cigarette use across waves. Weighted generalized estimating equation models adjusted for sociodemographic variables.

**Findings:**

Of never ENDS users at baseline, an estimated 1.7% reported initiating ENDS use by follow-up. Of former ENDS users, an estimated 12.1% relapsed into ENDS use. Of periodic ENDS users at baseline, 13% progressed to established ENDS use. Of baseline current ENDS users, 46.3% discontinued ENDS use. The corresponding transitions for cigarette smoking were 1.6% (initiation), 4.8% (relapse), 21.1% (progression), and 14% (discontinuation). Adults aged 18–24 (vs. older age), Hispanics (vs. non-Hispanic white), and past 12-month cannabis users were more likely to initiate ENDS or cigarettes (all *p* < 0.05). Having any internalizing mental health symptoms increased the odds of ENDS initiation, while externalizing symptoms increased the odds of cigarette initiation. Those who perceived nicotine as very harmful (vs. none/low harm) were more likely to discontinue ENDS. Current cigarette users (vs. non-users) at baseline were more likely to initiate, relapse, or discontinue ENDS (all *p* < 0.05) and vice versa.

**Interpretation:**

We observed high changeability in ENDS and cigarette use among US adults over time. In absolute terms, ENDS use grew, while smoking fell. Tobacco control programs should focus on priority populations, including young adults and people with internalizing and externalizing mental health symptoms.

**Funding:**

10.13039/100000002National Institutes of Health, R01-CA246606-01A1, R01-DA048390.

**Research in context**

**Evidence before this study**

We searched PubMed, Google Scholar, and medRxiv for longitudinal studies reporting on transitions in the use of electronic nicotine delivery systems (ENDS) and cigarettes among adults. We used the following search terms: (“cigarettes” OR “combustible cigarettes” OR “smoking” OR “tobacco smoking” OR “tobacco use”) AND (“electronic cigarettes” OR “e-cig” OR “ecig” OR “electronic nicotine delivery system” OR “ENDS” OR “vape” OR “vaping” OR “nicotine vaping products”) AND (“transitions” OR “behavioral transitions” OR “use pattern”). Our search was limited to longitudinal studies among adults which were published before November 1, 2022, without language limitation. We also included studies in the revision stage between November 1, 2022 and April 23, 2023. Our search identified 13 studies, primarily from the PATH survey (n = 12). Most of the studies focused on the initiation, cessation, and relapse of ENDS and cigarette use in the first three waves (2013–2016) of the PATH Study, and no study included progression as a transition outcome. The tobacco product landscape has rapidly evolved over the last several years, particularly for ENDS products. Therefore, it’s important to continuously monitor trends in ENDS and cigarette use transitions using the most up-to-date available data (2015–2019) and to identify the underlying drivers of these transitions within the changing tobacco product marketplace. This will help to inform public health policy, planning, and practice.

**Added value of this study**

The findings of this nationally representative longitudinal study add to the growing literature on the behavioral transitions in ENDS and cigarette use among US adults. Our results showed that discontinuing ENDS use was common among US adults. However, in absolute terms, ENDS use (including relapse) was growing, while smoking was falling. The current established ENDS use did not discourage relapse to cigarette smoking among former smokers, nor smoking initiation among never smokers. Conversely, current established cigarette smoking encouraged relapse to ENDS use among former users and initiation to vape among never ENDS users. The observed patterns of behavioral transitions in ENDS and cigarette use across the three most recent waves of PATH Study suggest surprisingly high changeability in ENDS and cigarette use among US adults. Adults aged 18–24 (vs. older age), Hispanics (vs. non-Hispanic white), and past 12-month cannabis users were more likely to initiate ENDS or cigarettes at follow-up. Internalizing mental health symptoms increased the odds of ENDS initiation, while externalizing symptoms increased the odds of cigarette initiation at follow-up. Those who perceived nicotine as very harmful (vs. none/low harm) were more likely to discontinue ENDS. Current cigarette users (vs. non-users) at baseline were more likely to initiate, relapse, or discontinue ENDS. Current ENDS users (vs. non-users) at baseline were more likely to initiate, relapse, or discontinue cigarettes by the follow-up.

**Implications of all the available evidence**

Our findings confirm the value of consistent monitoring of ENDS and cigarette use transitions, especially among people who are more vulnerable to these transitions. The findings of this nationally representative study of US adults provide evidence that can inform tobacco regulatory policies and cessation interventions. Although it is difficult to conclude that increasing initiation (and relapse) of ENDS use is the reason for decreasing cigarette use, ENDS may play a role in this decline. This needs to be examined in future longitudinal studies. Tobacco control programs should focus on priority populations including young adults, tobacco product users who co-use other substances (e.g., cannabis), and people with mental health conditions.

## Introduction

Many Americans use electronic nicotine delivery systems (ENDS, also known as e-cigarettes or vapes), with the majority vaping alongside smoking.^1^^,^^2^ Over 9.1 million (3.7%) US adults were current users of ENDS in 2020, and 30.8 million (12.5%) currently smoked cigarettes, according to the US Centers for Diseases Control and Prevention (CDC).^2^ The harms of smoking are well established, but the population benefits and harms of ENDS use are a matter of ongoing debate. Some propose that ENDS use is a valuable harm-reduction strategy that helps adults quit smoking combustible cigarettes.^3^, ^4^, ^5^ Others see ENDS use as a gateway to nicotine addiction or transition to cigarette smoking among naïve adolescents,^6^^,^^7^ and even a dangerous behavior due to emerging health hazards including lung damage.^8^

During the last several years, the tobacco product landscape has experienced a rapid series of changes, from the introduction of ENDS into the US in 2006 (e.g., cigalike),^9^^,^^10^ domination by high-tech JUUL in 2017,^11^ bans of ENDS flavors,^12^ new taxes,^13^^,^^14^ bans on the purchase of tobacco products under age 21,^15^ and most recently a proposed menthol cigarette ban.^16^ Many of these changes may affect behavioral transitions in ENDS and cigarette use.^17^, ^18^, ^19^ Furthermore, these changes may be unequally distributed across population subgroups, especially among vulnerable populations (young people, those with mental health conditions, or lesbian, gay, or bisexual (LGB) individuals). For example, people with mental health condistions are more likely to smoke cigarettes compared to those without mental health conditions.^20^, ^21^, ^22^ Emerging evidence also shows an association between ENDS use and mental health issues, including internalizing (e.g., feeling very trapped/sad/depressed) and externalizing (e.g., hard time paying attention) problems.^22^^,^^23^ Therefore, understanding these trends in behavioral transitions and how their distribution among vulnerable populations can inform public health policy, planning, and practice to reduce tobacco use.

Longitudinal studies have examined initiation, cessation, and relapse of tobacco product use.^17^^,^^18^^,^^24^, ^25^, ^26^ For example, using the first 3 waves (2013–2016) of the Population Assessment of Tobacco and Health (PATH) data, Stanton et al.^17^^,^^25^ found that the most typical usage pattern involved discontinued use, with about half (54%) of youth and 60% of all adults discontinuing ENDS use within 2 years at W2 and W3 or within 1 year at W3. In a similar study, Taylor et al.^18^ found that smoking remained persistent across time, regardless of age, with most smokers continuing to smoke at all three of these PATH waves. Room et al.’s^24^ examination of W3 and W4 PATH data on youth transitions in polytobacco use over time found that ENDS use may be the most common entry point to tobacco use. These studies provide important information about transitions in ENDS and cigarette use. However, the increasing diversification of ENDS products (including the emergence, rise, and fall of the novel fourth generation of ENDS products (e.g., JUUL)^27^ between 2015 and 2019 along with changes in tobacco control policies have implications for efforts to consistently monitor behavioral transitions and how ever and current ENDS or cigarette use can shape future behavioral transitions, including initiation, relapse, progression, and cessation.

Studies have also identified underlying drivers of behavioral transitions in ENDS and cigarette use. Correlates of the broad behavioral transitions of initiation,^28^ cessation^29^ and relapse^30^ were examined for the first three waves of PATH data. For example, older age and being gay or bisexual were associated with the initiation of tobacco use across products, and ENDS use was associated with some cigarette cessation behavior.^28^^,^^29^ Factors such as older age, being non-Hispanic black or Hispanic, and being bisexual were also associated with relapse.^30^ Continued evaluation of factors associated with behavioral transitions (including progression) in ENDS and cigarette use within the changing tobacco product marketplace is important to inform the development of strategies focused on populations at greater risk.

In this study, we analyzed data from W3 to W5 of the PATH Study to examine longitudinal transitions in ENDS and cigarette use behavior over approximately four years. We sought to assess whether never ENDS and cigarette users start using these products, former users relapse, periodic users progress to more established users, and current users discontinue use over time. We also examined predictors of these behavioral transitions for ENDS and cigarette use.

## Methods

### Participants and procedures

Data came from the PATH Study, a longitudinal, nationally representative study of the US non-institutionalized population ages 12 and older that tracks tobacco product use over time.^31^ The study was conducted by Westat and approved by their institutional review board. PATH used a four-stage stratified area probability sample design, varying sampling rates for adults by age, race, and tobacco use status. Adults (ages 18+) were screened in person and African Americans, 18–24 years old, and tobacco users were oversampled.^32^ Participants provided informed consent, completed surveys via computer-assisted self-report either in English or Spanish, and received $35 for completing each survey. Further details on the study design and methods are available elsewhere.^32^^,^^33^ The datasets generated and analyzed during the current study are available from the PATH study Public-Use Files (https://doi.org/10.3886/ICPSR36498.v17). Our study follows relevant reporting standards on Enhancing the Quality and Transparency of Health Research.^34^

The current study focused on adults but not youth because they have distinct patterns of tobacco product use. We included the most recent three waves of the adults’ PATH data: Wave (W) 3 (n = 28,146; October 2015–October 2016), W4 (n = 33,821; December 2016–January 2018); and W5 (n = 34,309; December 2018–November 2019). The W3, W4, and W5 weighted adult interview response rates for the W1 Cohort were 78.4%, 73.5%, and 69.4%, respectively. For more details on wave response rates, see PATH study Public-Use User Guide (https://doi.org/10.3886/ICPSR36498.v17).

We created an analytic sample for adults who completed the study from W3–W5, and then cohorts for each outcome for ENDS and cigarettes. The analytic samples include person-intervals for ENDS: initiation (n = 23,985), relapse (n = 14,210), progression (n = 3044), and cessation (n = 4492); and for cigarettes: initiation (n = 11,845), relapse (n = 15,156), progression (n = 4632), and cessation (n = 16,147) (see Supplementary Fig. S1 for details).

We also created analytic samples for adults who completed the study from W3–W4 (n = 25,052) and W4–W5 (n = 28,677) (Table 1). The W4–W5 sample was larger because 7972 adults had aged-up (from the youth cohort) or were newly added to W4 as a new cohort (Supplementary Fig. S1). To support examination of behavioral transitions (initiation, relapse, progression, and cessation; see below for definitions) in ENDS and cigarette use without excluding the replenishment sample (n = 7972), we created four cohorts for each product in each timeframe resulting in 8 cohorts as described in Supplementary Fig. S1 (i.e., W3–W4 and W4–W5). For example, for the most recent period (W4–W5), the cohorts consisted of never users at W4 who were at risk of initiation (n = 14,556 for ENDS, n = 7895 for cigarettes) at W5, former users at risk of relapse (n = 8747 for ENDS, n = 9656 for cigarettes), ever users (including current experimental users) at risk of progression (n = 1653 for ENDS, n = 2746 for cigarettes) to current established users (including from some days to daily users) at W5. The last group was current established or experimental users at W4 (n = 2431 for ENDS, n = 9087 for cigarettes) who were prone to discontinue (hereafter cessation) at W5.

**Table 1:** Baseline demographic characteristics by exposure waves, PATH Study.

Characteristics	W3 (*N* = 25,052)		W4 (*N* = 28,677)	
	n	Wt% (95% CI)	n	Wt% (95% CI)
**Age group (years)**				
18–24	7426	12.1 (11.7–12.5)	9264	11.8 (11.4–12.2)
25–44	8777	33.9 (33.2–34.5)	9763	33.6 (33.0–34.2)
45–64	6489	34.8 (34.2–35.3)	7045	35.0 (34.4–35.8)
65+	2358	19.6 (18.8–19.8)	2602	19.6 (19.0–20.1)
**Sex**				
Male	12,086	47.4 (46.8–48.0)	13,863	48.0 (47.0–48.3)
Female	12,966	52.6 (51.9–53.2)	14,814	52.0 (51.7–53.0)
**Sexual orientation**				
Straight	22,764	93.0 (92.6–93.5)	25,881	93.0 (92.5–93.5)
LGB	1969	5.0 (4.6–6.3)	2517	5.7 (5.3–6.1)
Not reported	319	2.0 (1.7–2.3)	279	1.3 (1.1–1.5)
**Race/ethnicity**				
Non-Hispanic White	14,570	65.1 (64.5–65.7)	16,288	64.9 (64.3–65.5)
Non-Hispanic Black	3836	11.7 (11.3–12.1)	4466	11.9 (11.5–12.3)
Hispanic	4735	15.4 (15.0–15.8)	5686	15.5 (15.0–15.9)
Other races	1911	7.8 (7.5–8.1)	2237	7.7 (7.4–8.0)
**Education**				
Less than high school	4862	16.0 (15.6–16.5)	5304	15.7 (15.2–16.2)
High school graduate	5935	22.7 (22.2–23.2)	6800	22.1 (21.5–22.7)
Some college	8720	31.9 (31.3–32.5)	10,206	32.1 (31.5–32.7)
Bachelor’s degree or more	5430	29.4 (28.8–29.9)	6253	30.1 (29.6–30.7)
**Income**^a^				
Less than $25,000	8952	27.7 (26.9–28.6)	9414	25.4 (24.6–26.2)
$25,000–$49,999	5456	21.0 (20.2–21.8)	6325	21.0 (20.2–21.8)
$50,000–$99,999	5438	24.9 (24.1–25.7)	6538	25.4 (24.5–26.3)
$100,000 or more	3766	19.5 (18.6–20.4)	4819	21.8 (20.9–22.7)
Not reported	1440	6.9 (6.4–7.4)	1581	6.4 (5.9–6.9)
**Internalizing symptoms**				
No	17,079	74.7 (74.0–75.5)	18,369	72.1 (71.2–72.9)
Yes	7918	25.3 (24.5–26.0)	10,261	27.9 (27.1–28.8)
**Externalizing symptoms**				
No	24,026	97.4 (97.1–97.6)	27,030	96.3 (96.0–96.6)
Yes	979	2.6 (2.4–2.9)	1603	3.7 (3.4–4.0)
**Current use of ENDS**				
No	23,450	96.7 (96.5–96.7)	27,012	96.9 (96.6–97.1)
Yes	1551	3.3 (3.0–3.5)	1644	3.1 (2.9–3.4)
**Current use of cigarettes**				
No	17,110	82.1 (81.5–82.7)	20,378	82.6 (81.9–83.2)
Yes	7918	17.9 (17.3–18.5)	8271	17.4 (16.8–18.0)
**Current other tobacco use**				
No	22,451	94.2 (93.9–94.5)	25,842	94.0 (93.7–94.3)
Yes	2601	5.8 (5.5–6.1)	2835	6.0 (5.7–6.3)
**Past 12 months cannabis use**^c^				
No	18,837	92.4 (91.7–93.0)	18,905	88.6 (87.8–89.3)
Yes	2606	7.6 (7.0–8.3)	4434	11.4 (10.6–12.2)
**Nicotine dependence, ENDS**^d^	1482	20.8 (19.4–22.1)	1296	23.6 (21.5–25.7)
**Nicotine dependence, cigarettes**^d^	6074	44.7 (43.8–45.5)	7154	43.5 (42.8–44.2)
**Nicotine harm perception**				
None or little harm	6104	16.9 (16.3–17.5)	6612	16.5 (15.9–17.2)
Very harmful	18,853	83.1 (82.5–83.7)	21,993	83.5 (82.8–84.1)
**ENDS absolute harm perception**				
Non or little harm	11,291	37.2 (36.1–38.2)	12,276	34.5 (33.6–35.4)
Very harmful	13,312	62.8 (61.8–63.9)	16,041	65.5 (64.6–66.4)
**Cigarettes absolute harm perception**^b^				
None or little harm	3552	9.0 (8.6–9.5)	4028	9.7 (9.1–10.2)
Very harmful	21,437	91.0 (90.5–91.4)	24,583	90.3 (89.7–90.8)
**Comparative harm perception**				
Less harmful	6908	24.0 (23.3–24.8)	7077	20.8 (20.0–21.6)
Equally or more harmful	17,744	73.8 (73.0–74.6)	21,254	77.8 (76.9–78.6)
Don’t know	400	2.2 (1.9–2.5)	346	1.4 (1.2–1.7)

The reported numbers are unweighted. Weighted percentages were calculated using W3 single wave weights (for W3) and W4 single wave weights (for W4).

W = wave, Wt% = weighted percentage, CI = confidence interval, LGB = Lesbian, Gay, bisexual, or something else, ENDS = electronic nicotine delivery system.

a Amounts is in US dollars. Because the income question referred to the past 12 months, we used the succeeding waves information.

b Vaping harm perception relative to cigarettes.

c n = 21,443 at W3 and n = 23,339 at W4 provided information on the past 12-month cannabis use.

d Nicotine dependence (weighted mean and 95% CI) for ENDS and cigarettes were calculated for adult established or experimental users who responded to 16 dependency items for each product at each exposure time point.

### Measures

#### Behavioral transitions

The study looked at longitudinal transitions between survey waves, in four outcomes. The first outcome was an *initiation* in use of ENDS or cigarettes, defined as the transition from never use to ever use. The second outcome was *relapse*, defined as the transition from former use to current use of any type (experimental or established). The third outcome was *progression* in ENDS or cigarette use, defined as transition from current experimental use to established use (either somedays or daily) or established use on some days to daily established use. The fourth outcome was *cessation* in use of ENDS or cigarettes, defined as transition from current established or current experimental use to former use. For these four outcomes, we coded yes as 1, and no as 0. See definitions in Supplementary Table S2.

#### Demographic characteristics

In line with previous literature,^28^, ^29^, ^30^^,^^35^, ^36^, ^37^, ^38^ several sociodemographics were included at baseline (W3 or W4): age (18–24, 25–44, 45–64, or 65+ years), sex (male or female), sexual orientation (lesbian, gay, bisexual, something else (LGB) or straight), race/ethnicity (non-Hispanic white, non-Hispanic black, Hispanic, or other races (i.e., all other races, more than one race), annual household income (less than $25,000, $25,000–$49,999, $50,000–$99,999, or $100,000 or more), and educational attainment (less than high school or GED, high school graduate, some college, bachelor’s degree or more).

Missing data on sex, race, and ethnicity were imputed by the PATH Study as described in the PATH Public-Use Files User Guide (https://doi.org/10.3886/ICPSR36498.v17). In brief, imputations were used for both the Wave 1 and Wave 4 cohorts because respondents in Wave 4 came from two different samples chosen at different times: the replenishment sample and the Wave 1 Cohort. The method for imputing missing data differed for these two groups. For the replenishment sample, imputation involved using information from the Wave 4 household screener and then applying statistical imputation methods as described in the PATH Public-Use Files User Guide. For the Wave 1 Cohort, imputed values from Wave 1 were also used, along with new statistical imputation methods and information from previous waves. Although members of the Wave 1 Cohort who were not in the Wave 4 Cohort were excluded from the Wave 4 Cohort cross-sectional weighting process, they were still included in the imputation process to ensure that imputed variables were available for all Wave 4 respondents. More details on imputation can be found in section 5.3.2 describes in Public-Use Files User Guide updated for Wave 5 (https://www.icpsr.umich.edu/files/NAHDAP/documentation/ug36498-all.pdf). For education, missing values were <1% and were excluded from the analysis. Annual household income was added from subsequent timepoint since it asks participants’ income in the past 12 months. Also, respondents with missing income or sexual orientation information may not be a random subset of population-based survey participants and may differ on other relevant sociodemographic characteristics.^39^ Hence, for sexual orientation and income variables reported as “do not know” or “missing”, we categorized them as “not reported.”

#### Mental health

Self-reported mental health status was assessed separately for internalizing and externalizing symptoms via the Global Appraisal of Individual Needs—Short Screener (GAIN–SS), modified for the PATH Study. We assessed internalizing symptoms using four items and externalizing symptoms using seven items (See Supplementary Table S3 for GAIN–SS items). The response options for both internalizing and externalizing problems were coded on a scale of 0 (never) to 3 (past month), regardless of the type of problem. Following the previous study^23^ and to simplify the presentation of the results, analyses grouped participants into two categories: those who never reported any problems (coded as 0) and those who reported at least one problem in the past 12 months or more (coded as 1).

#### Tobacco products and cannabis use

ENDS and cigarettes use definitions are in Supplementary Table S1. Current use of other tobacco products (OTPs) was defined as ever use of traditional cigar, cigarillo, filtered cigar, pipe, hookah, snus pouch or other smokeless tobacco fairly regularly, and currently use them every day or on some days. For cannabis use, participants were asked “*In the past 12 months, have you used marijuana, hash, THC, grass, pot or weed?*” with yes or no answers.

#### Nicotine dependence

We used the validated 16-item nicotine dependence scale; the 5-point response scale ranged from “not at all true of me” to extremely true of me” (with one dichotomous item that was scored 1 or 5).^40^ In line with previous studies,^40^^,^^41^ we scored the nicotine dependence measure by averaging the items. Nicotine dependence for ENDS and cigarettes were collected only from current users of these products.^33^

#### Harm perception

Comparative harm perception was assessed by asking whether using ENDS is less harmful, about the same, or more harmful than smoking cigarettes, which we categorized as less vs. equal or more harmful.^42^^,^^43^ Perceived nicotine harm was assessed by asking how harmful nicotine is to participants’ health, which was classified as none or little harm (not at all harmful, slightly harmful, somewhat harmful) vs. very harmful (very harmful, extremely harmful). Absolute perceived harm of cigarettes and ENDS were assessed by asking how harmful smoking cigarettes/using ENDS is to participants’ health, which we categorized in the same way.

### Data analysis

First, we characterized the sample. We reported unweighted sample frequencies and weighted sample percentages, sample means, and estimated population frequency for US adults. Weighting was based on single or replicated weights to account for the sampling scheme and create nationally representative estimates of US adults who experienced behavioral transitions in ENDS or cigarette use. The PATH Study single and all-waves weights were used to compute longitudinal projections as described in PATH Study Public-Use Files User Guide (https://doi.org/10.3886/ICPSR36498.v17). These projections, which were adjusted for population size, are an accurate representation of the non-institutionalized adult population aged 18 and above residing in the US, except for those who were incarcerated. Variances were estimated using balanced repeated replication method with Fay adjustment (e.g., *Fay* = 0.3).^33^^,^^44^ To visualize a flow in transition rates in ENDS and cigarette use from one timepoint of values to another, we created the Sankey diagram using SankeyMATIC (https://sankeymatic.com/).

Second, we examined correlates of behavioral transitions (initiation, relapse, progression, or cessation) in cigarettes and ENDS use from W3–W5. We conducted separate weighted generalized estimating equation (GEE)^28^ analyses for each transition outcome. GEE examines changes over time, while accounting for correlation among observations contributed by the same individuals.^28^^,^^45^ The GEE models used unstructured covariance and within-person correlation matrices and a binomial distribution using the logit link function. Analyses controlled for demographics, internalizing and externalizing mental health symptoms, current tobacco products use, cannabis use, nicotine dependence, and harm perception. Analyses also controlled for ENDS use in models for cigarette transitions and vice versa. The models used W5 all-waves weights to produce nationally representative estimates. Variance computations used the balanced repeated replication method as recommended by the PATH Study. We report the findings in the main paper as adjusted odds ratios (AOR) and corresponding 95% confidence intervals (CIs). These GEE models did not include the replenishment samples that the PATH Study added at W4.

Third, we examined correlates of transitions in analyses that also included adults who were newly added to W4. These additional participants aged-up into the adult cohort from the youth cohort or were new participants that the PATH Study recruited for W4 (n = 7972; see Supplementary Fig. S1). The new data structure required separating the analyses of change to be for W3–W4 and W4–W5. These analyses used weighted multivariable logistic regression models that controlled for the same correlates noted previously. We report these findings in Supplementary Tables S4–S7.

Fourth, we computed cross-tabulation combinations of ENDS and cigarette use for W3–W4 and W4–W5 using four use status i) exclusive established ENDS use, ii) exclusive established cigarette use, iii) dual established use of ENDS and cigarettes, and iv) no-use. To examine potential collinearity among predictors in our models, we looked for tolerance >0.1, variance inflation <10, and eigenvalues not close to zero, and found no obvious collinearity. All analyses were performed using SAS 9.3. We set the statistical significance level at α = 0.05. The results of the GEE model appear in the main tables, and results for multivariable regression models appear in the Supplementary materials.

### Ethical approval

PATH data collection was approved by Westate’s Institutional Review Board with written informed consent that was obtained from participants. This work has used publically available de-identified data that does not deal with any individual or personal sensitive data. Hence, the current study does not require any ethical permissions.

### Role of the funding source

The National Institutes of Health under awards R01-CA246606-01A1 and R01DA048390 supported MEK’s and NTB time working on the paper. The funders had no role in the study design, data collection and analysis, preparation of this manuscript, interpretation of results, or the decision to submit the manuscript for publication.

## Results

### ENDS use transitions

#### Initiation

**Fig. 1:**
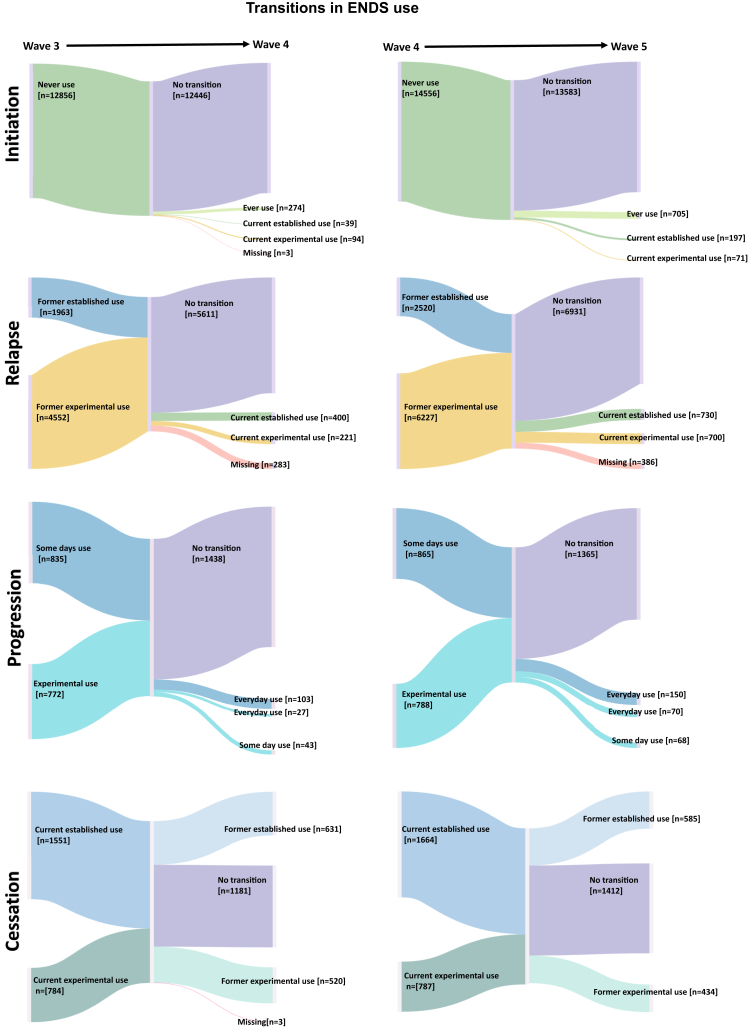
Longitudinal transitions in ENDS use among US adults from W3–W4 (left 2 columns) and W4–W5 (right 2 columns), PATH Study.

Of the 12,856 adult never ENDS users, a population estimate of 1.7% reported initiating ENDS use from W3–W5 (Fig. 1 and Table 2). ENDS use initiation was less likely among older adults than young adults (AOR range = 0.04–0.25, Table 3). Initiation was more likely among Hispanic (vs. white, AOR = 1.44; 95% CI: 1.07–1.95) and less likely among other races (vs. white, AOR = 0.66; 95% CI: 0.46–0.94). Initiation also was more likely to occur among adults who were experiencing internalizing symptoms (vs. no symptom, AOR = 1.78; 95% CI: 1.39–2.29), current cigarette users (vs. non-current users; AOR = 5.57; 95% CI: 4.15–7.48), current other tobacco products users (vs. non-current users; AOR = 2.53; 95% CI: 1.76–3.66), and past 12-month cannabis user (vs. non-users; AOR = 2.99; 95% CI: 2.22–4.02) from W3–W5 (Table 3).

**Table 2:** Estimated number of behavioral transitions in ENDS and cigarette use among US adults over Waves 3–5, PATH Study, 2015–2019.

Products	Behavioral transitions (total number)	n (transitioned)	Wt% (95% CI)	Estimated No. of US population experienced transition	Ratio of initiation or relapse vs. cessation
ENDS	Initiation (n = 23,985)	964	1.7 (1.6–1.9)	5,946,793	1.4
	Relapse (n = 14,210)	1694	12.1 (11.4–12.8)	8,300,946	
	Progression (n = 3044)	356	13.0 (11.4–14.6)	1,755,929	
	Cessation (n = 4492)	1939	46.3 (44.4–48.2)	9,857,799	
Cigarettes	Initiation (n = 11,845)	468	1.6 (14–1.9)	2,421,155	0.8
	Relapse (n = 15,156)	1472	4.8 (4.5–5.2)	8,020,106	
	Progression (n = 4632)	1062	21.1 (19.7–22.6)	5,338,237	
	Cessation (n = 16,147)	2241	14.0 (13.3–14.8)	12,473,618	

W = wave, Wt% = weighted percentage, CI = confidence interval, ENDS = electronic nicotine delivery system.

Weighted percentages and CIs, and estimated numbers were calculated using PATH Wave 5 all-wave weights.

**Table 3:** Correlates of behavioral transitions in ENDS use over Waves 3–5, PATH Study.

Characteristics	ENDS use transitions							
	Initiation (n = 12,856)		Relapse (n = 14,210)		Progression (n = 3044)		Cessation (n = 4492)	
	Crude n/number at risk (Wt%)	Adjusted OR	Crude n/number at risk (Wt%)	Adjusted OR	Crude n/number at risk (Wt%)	Adjusted OR	Crude n/number at risk (Wt%)	Adjusted OR
**Overall**	964 (1.7)	─	1694 (12.1)	─	356 (13.0)	─	1939 (46.3)	─
**Age group**								
18–24^a^	490 (7.6)	Ref	736 (17.0)	Ref	168 (15.2)	Ref	788 (46.6)	Ref
25–44	283 (2.1)	**0.25 (0.19–0.32)**	714 (11.9)	**0.56 (0.46–0.69)**	127 (12.4)	0.89 (0.54–1.46)	703 (48.6)	0.87 (0.63–1.20)
45–64	161 (1.2)	**0.14 (0.10–0.19)**	215 (8.2)	**0.38 (0.28–0.51)**	52 (10.3)	0.60 (0.35–1.04)	383 (43.8)	0.76 (0.52–1.11)
65+	30 (0.3)	**0.04 (0.03–0.07)**	29 (7.0)	**0.39 (0.23–0.66)**	11 (16.8)	0.70 (0.29–1.69)	65 (44.2)	1.06 (0.56–2.02)
**Sex**								
Female^a^	469 (1.5)	Ref	848 (11.7)	Ref	150 (11.7)	Ref	975 (48.1)	Ref
Male	494 (2.0)	1.18 (0.93–1.51)	846 (12.3)	0.88 (0.73–1.06)	206 (14.0)	1.16 (0.79–1.70)	964 (44.9)	0.95 (0.72–1.25)
**Sexual Orientation**								
Straight^a^	854 (1.7)	Ref	1418 (11.5)	Ref	295 (12.7)	Ref	1661 (46.6)	Ref
LGB	108 (4.3)	1.28 (0.82–2.00)	260 (16.7)	1.18 (0.91–1.53)	59 (14.0)	1.15 (0.64–2.06)	267 (44.9)	0.91 (0.61–1.38)
Not reported	2 (0.1)	**0.14 (0.02–0.97)**	16 (13.8)	1.48 (0.69–3.18)	2 (14.3)	2.88 (0.42–19.53)	11 (31.3)	1.37 (0.27–7.06)
**Race/ethnicity**								
Non-Hispanic White	484 (1.5)	Ref	1040 (12.4)	Ref	240 (14.4)	Ref	1182 (44.0)	Ref
Non-Hispanic Black	182 (2.4)	1.29 (0.95–1.75)	180 (10.4)	0.97 (0.71–1.34)	37 (12.0)	0.93 (0.48–1.77)	244 (53.4)	0.88 (0.53–1.45)
Hispanic	219 (2.5)	**1.44 (1.07–1.95)**	310 (11.1)	1.07 (0.84–1.36)	48 (7.5)	**0.29 (0.16–0.54)**	372 (55.6)	1.29 (0.86–1.93)
Other races	79 (1.4)	**0.66 (0.46–0.94)**	164 (13.9)	1.23 (0.91–1.68)	31 (12.9)	0.71 (0.32–1.57)	141 (42.5)	0.88 (0.52–1.51)
**Educational**								
Less than high school	166 (2.0)	Ref	322 (12.1)	Ref	67 (9.3)	Ref	452 (51.7)	Ref
High school graduate	260 (1.9)	0.95 (0.67–1.34)	421 (12.7)	1.06 (0.80–1.41)	92 (13.0)	1.68 (0.90–3.12)	483 (45.7)	1.16 (0.78–1.74)
Some college	387 (2.2)	1.10 (0.80–1.53)	735 (13.0)	1.21 (0.95–1.55)	150 (13.5)	1.42 (0.81–2.48)	789 (45.2)	1.16 (0.82–1.65)
Bachelor’s degree or more	150 (1.1)	0.73 (0.48–1.12)	212 (9.3)	1.03 (0.75–1.41)	46 (16.5)	**2.33 (1.22–4.46)**	203 (43.6)	0.91 (0.56–1.47)
**Income**								
Less than $25,000^a^	357 (2.3)	Ref	662 (13.1)	Ref	132 (11.3)	Ref	854 (50.8)	Ref
$25,000–$49,999	206 (1.9)	1.18 (0.89–1.57)	410 (12.1)	1.01 (0.81–1.26)	84 (13.1)	0.72 (0.42–1.24)	439 (44.3)	0.85 (0.60–1.19)
$50,000–$99,999	190 (1.5)	1.07 (0.77–1.48)	346 (11.2)	0.95 (0.73–1.23)	86 (15.5)	0.92 (0.55–1.54)	369 (44.4)	0.88 (0.63–1.22)
$100,000 or more	154 (1.5)	1.24 (0.84–1.83)	200 (11.0)	0.96 (0.72–1.29)	37 (14.3)	0.72 (0.38–1.39)	179 (39.6)	0.70 (0.46–1.07)
Not reported	57 (1.2)	1.00 (0.58–1.73)	76 (12.5)	0.79 (0.52–1.19)	17 (11.6)	0.63 (0.20–1.98)	98 (48.0)	0.68 (0.36–1.28)
**Internalizing symptoms**								
No	561 (1.3)	Ref	818 (10.1)	Ref	177 (11.8)	Ref	1043 (46.5)	Ref
Yes	403 (3.3)	**1.78 (1.39–2.29)**	874 (14.8)	**1.30 (1.09–1.56)**	178 (14.1)	1.13 (0.76–1.68)	890 (46.2)	1.11 (0.85–1.47)
**Externalizing symptoms**								
No	900 (1.7)	Ref	1511 (11.6)	Ref	319 (12.8)	Ref	1781 (46.1)	Ref
Yes	64 (5.5)	1.18 (0.75–1.85)	181 (18.8)	1.32 (0.99–1.74)	36 (14.3)	1.16 (0.62–2.18)	151 (48.9)	1.59 (0.96–2.63)
**Current use of cigarettes**								
No	722 (1.4)	Ref	672 (9.1)	Ref	133 (14.1)	Ref	759 (40.1)	Ref
Yes	242 (6.6)	**5.57 (4.15–7.48)**	1021 (15.0)	**2.34 (1.88–2.92)**	222 (12.3)	0.86 (0.54–1.37)	1180 (51.3)	**1.55 (1.18–2.02)**
**Current other tobacco use**								
No	865 (1.6)	Ref	1365 (11.3)	Ref	281 (13.9)	Ref	1449 (44.8)	Ref
Yes	99 (5.1)	**2.53 (1.76–3.66)**	329 (17.4)	**1.49 (1.17–1.89)**	75 (9.7)	0.63 (0.37–1.06)	490 (52.2)	1.16 (0.80–1.68)
**Past 12-month cannabis use**^b^								
No	618 (1.3)	Ref	802 (9.4)	Ref	174 (12.8)	Ref	1007 (45.1)	Ref
Yes	161 (6.1)	**2.99 (2.22–4.02)**	288 (12.9)	**1.31 (1.06–1.61)**	52 (14.3)	1.12 (0.72–1.74)	269 (43.1)	0.81 (0.59–1.11)
**Nicotine harm perception**								
None or little harm	218 (3.5)	Ref	582 (13.8)	Ref	172 (14.9)	Ref	816 (38.3)	Ref
Very harmful	741 (1.5)	0.79 (0.60–1.05)	1106 (11.3)	0.99 (0.82–1.19)	183 (11.5)	0.75 (0.51–1.09)	1111 (54.4)	**1.50 (1.15–1.97)**
**ENDS absolute harm perception**								
None or little harm	431 (2.6)	Ref	1033 (13.8)	Ref	291 (13.8)	Ref	1436 (43.3)	Ref
Very harmful	521 (1.4)	0.86 (0.67–1.10)	641 (10.1)	**0.75 (0.63–0.90)**	62 (9.7)	0.74 (0.42–1.32)	474 (60.3)	1.32 (0.91–1.93)
**Comparative harm perception**								
Less harmful	246 (2.3)	Ref	577 (13.8)	Ref	205 (16.3)	Ref	905 (38.9)	Ref
Equally or more harmful	703 (1.6)	0.91 (0.69–1.19)	1098 (11.3)	0.75 (0.63–0.90)	148 (9.7)	0.66 (0.43–1.02)	1016 (56.9)	1.31 (0.98–1.75)
**ENDS nicotine dependence**^c^	─	─	─	─	16.7 (1.6)^c^	**1.03 (1.02–1.04)**	13.9 (0.7)^c^	**0.97 (0.96–0.98)**

W = wave, Wt% = weighted percentage, CI = confidence interval, LGB = Lesbian, Gay, bisexual, or something else, ENDS = electronic nicotine delivery system, Ref = reference.

All analyses were weighted using PATH Wave 5 all-wave weights. Bold-faced indicates *p* < 0.05.

a Unweighted *n* and weighted row percentages.

b Missing for cannabis use was >5% within transition outcomes.

c Nicotine dependence shows the weighted mean and standard error among individuals who progressed or discontinued using ENDS.

#### Relapse

Of the 14,210 adults former ENDS users, a population estimate of 12.1% reported relapse in ENDS use from W3–W5 (Fig. 1 and Table 2). Relapse in ENDS use was less likely among older age groups than young adults (AOR range = 0.39–0.56, Table 3). Relapse was more likely among adults who were experiencing internalizing symptoms (vs. no symptom, AOR = 1.30; 95% CI: 1.09–1.56), current cigarette users (vs. non-current users; AOR = 2.34; 95% CI: 1.88–2.92), current other tobacco products users (vs. non-current users; AOR = 1.49; 95% CI: 1.17–1.89), and past 12-month cannabis user (vs. non-users; AOR = 1.31; 95% CI: 1.06–1.61) (Table 3). Perceiving ENDS as very harmful (vs. none or little harm; AOR = 0.75; 95% CI: 0.63–0.90) was associated with lower odds of relapse in ENDS use from W3–W5 (Table 3).

#### Progression

Of the 3044 adult periodic ENDS users, a population estimate of 13% reported progression in ENDS use from W3–W5 (Fig. 1 and Table 2). Progression in ENDS use was less likely among Hispanic (vs. white, AOR = 0.29; 95% CI: 0.16–0.54). Adults with bachelor’s degree or more (vs. less than high school; AOR = 2.22; 95% CI: 1.22–4.46) and high nicotine dependence rate (AOR = 1.03; 95% CI: 1.02–1.04) were more likely to progress in ENDS use (Table 3).

#### Cessation

Of the 4492 adults current experimental or established ENDS users, a population estimate of 46.3% reported cessation in ENDS use from W3–W5 (Fig. 1 and Table 2). Cessation in ENDS use was less likely among Hispanic (vs. white, AOR = 0.29; 95% CI: 0.16–0.54). Adults with bachelor’s degree or more (vs. less than high school; AOR = 2.22; 95% CI: 1.22–4.46) and high nicotine dependence rate (AOR = 1.03; 95% CI: 1.02–1.04) were more likely to discontinue ENDS use (Table 3).

### Cigarette use transitions

#### Initiation

**Fig. 2:**
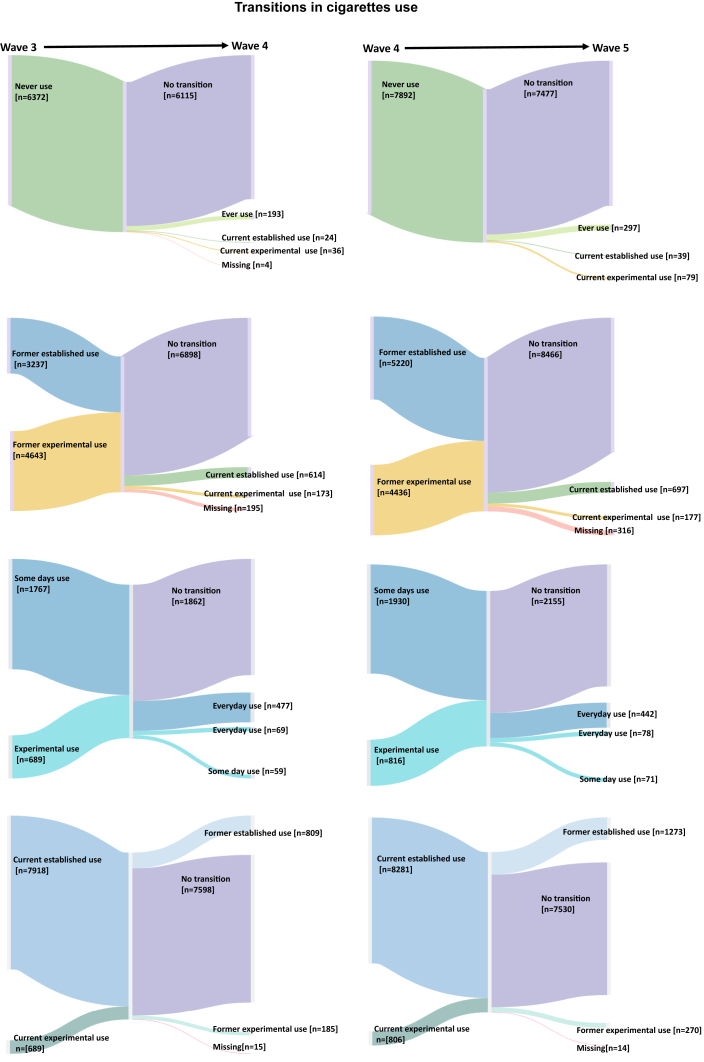
Longitudinal transitions in cigarettes use among US adults from W3–W4 (left 2 columns) and W4–W5 (right 2 columns), PATH Study.

Of the 11,845 adult never cigarette smokers, a population estimate of 1.6% reported initiating cigarette smoking over W4–W5 (Fig. 2 and Table 2). Cigarette smoking initiation was less likely among adults aged 25–44 than young adults (AOR = 0.17; 95% CI: 0.11–0.26, Table 4). Smoking initiation was more likely among males (vs. females, AOR = 1.74; 95% CI: 1.17–2.58). Hispanic (vs. white, AOR = 1.58; 95% CI: 1.03–2.42), those who were experiencing externalizing symptoms (vs. no symptom, AOR = 1.95; 95% CI: 1.04–3.65), current ENDS users (vs. non-current users; AOR = 5.47; 95% CI: 1.75–17.14), current other tobacco users (vs. non-current users; AOR = 2.29; 95% CI: 1.04–5.01), past 12-month cannabis use (AOR = 2.14; 95% CI: 1.50–3.06). Adults who made an annual income of $50,000–$99,999 (vs. <$25,000; AOR = 0.40; 95% CI: 0.24–0.66) were less likely to initiate smoking cigarette (Table 4).

**Table 4:** Correlates of behavioral transitions in cigarette use over Waves 3–5, PATH Study.

Characteristics	Cigarette use transitions							
	Initiation (n = 11,845)		Relapse (n = 15,156)		Progression (n = 4632)		Cessation (n = 16,147)	
	Crude n/number at risk (Wt%)^a^	Adjusted OR	Crude n/number at risk (Wt%)^a^	Adjusted OR	Crude n/number at risk (Wt%)^a^	Adjusted OR	Crude n/number at risk (Wt%)^a^	Adjusted OR
**Overall**	468 (1.6)	─	1472 (4.8)	─	1062 (21.1)	─	2241 (14.0)	─
**Age group**								
18–24	415 (6.0)	Ref	513 (15.3)	Ref	320 (19.9)	Ref	688 (24.4)	Ref
25–44	53 (0.7)	**0.17 (0.11–0.26)**	634 (7.7)	**0.63 (0.50–0.78)**	430 (19.6)	0.97 (0.69–1.37)	954 (14.6)	**0.56 (0.45–0.69)**
45–64	d	d	265 (3.0)	**0.28 (0.21–0.36)**	261 (21.3)	0.89 (0.60–1.31)	458 (9.7)	**0.45 (0.36–0.57)**
65+	d	d	60 (1.0)	**0.08 (0.05–0.12)**	51 (20.0)	0.64 (0.33–1.25)	141 (15.3)	0.79 (0.58–1.08)
**Sex**								
Female	202 (1.2)	Ref	730 (4.6)	Ref	527 (23.1)	Ref	1054 (13.6)	Ref
Male	266 (2.3)	**1.74 (1.17–2.58)**	742 (5.0)	0.89 (0.73–1.08)	535 (19.6)	0.84 (0.64–1.11)	1187 (14.4)	1.04 (0.88–1.23)
**Sexual Orientation**								
Straight	405 (1.5)	Ref	1273 (4.5)	Ref	893 (20.8)	Ref	1965 (14.0)	Ref
LGB	58 (4.3)	1.38 (0.82–2.33)	187 (10.0)	1.13 (0.82–1.55)	153 (24.0)	1.10 (0.69–1.76)	248 (15.0)	0.80 (0.60–1.07)
Not reported	5 (1.2)	2.76 (0.94–8.06)	12 (4.9)	1.29 (0.45–3.70)	16 (16.7)	0.63 (0.15–2.60)	28 (11.8)	1.03 (0.49–2.19)
**Race/ethnicity**								
Non-Hispanic white	219 (1.3)	Ref	816 (3.9)	Ref	557 (23.5)	Ref	1291 (13.5)	Ref
Non-Hispanic black	68 (1.5)	1.09 (0.68–1.75)	204 (7.8)	1.29 (0.97–1.72)	211 (21.1)	0.72 (0.48–1.08)	312 (11.0)	0.68 (0.53–0.87)
Hispanic	133 (2.3)	**1.58 (1.03–2.42)**	343 (7.8)	1.05 (0.81–1.36)	206 (16.2)	**0.51 (0.36–0.74)**	473 (19.0)	1.05 (0.83–1.33)
Other races	48 (2.1)	1.73 (0.74–4.06)	109 (5.1)	0.99 (0.67–1.44)	88 (19.8)	0.70 (0.39–1.27)	165 (17.2)	1.01 (0.74–1.40)
**Educational**								
Less than high school	59 (2.2)	Ref	307 (7.7)	Ref	314 (26.6)	Ref	493 (10.8)	Ref
High school graduate	148 (1.7)	0.61 (0.32–1.14)	338 (5.8)	0.77 (0.58–1.03)	279 (21.7)	0.69 (0.46–1.02)	514 (11.1)	0.91 (0.72–1.14)
Some college	227 (2.2)	0.67 (0.36–1.25)	579 (5.3)	**0.59 (0.46–0.76)**	360 (21.0)	**0.63 (0.43–0.92)**	863 (16.0)	**1.28 (1.04–1.57)**
Bachelor’s degree or more	33 (0.8)	0.50 (0.20–1.23)	241 (2.7)	**0.49 (0.36–0.67)**	104 (13.5)	**0.45 (0.27–0.75)**	360 (23.9)	**1.92 (1.47–2.51)**
**Income**								
Less than $25,000	187 (2.5)	Ref	581 (8.2)	Ref	539 (24.5)	Ref	921 (11.7)	Ref
$25,000–$49,999	85 (1.6)	0.77 (0.43–1.37)	343 (5.4)	1.04 (0.60–1.81)	237 (22.4)	0.89 (0.48–1.66)	491 (13.2)	1.03 (0.83–1.29)
$50,000–$99,999	70 (0.8)	**0.40 (0.24–0.66)**	310 (4.2)	0.86 (0.50–1.49)	160 (18.1)	0.74 (0.38–1.43)	453 (16.0)	1.05 (0.84–1.31)
$100,000 or more	87 (1.5)	0.86 (0.49–1.48)	176 (2.4)	**0.53 (0.30–0.93)**	77 (14.9)	0.68 (0.33–1.41)	260 (24.5)	**1.57 (1.17–2.10)**
Not reported	57 (1.2)	0.73 (0.39–1.35)	62 (3.6)	0.66 (0.38–1.14)	49 (16.8)	0.92 (0.49–1.72)	116 (14.2)	1.19 (0.80–1.77)
**Internalizing symptoms**								
No	286 (1.3)	Ref	867 (4.0)	Ref	607 (20.2)	Ref	1321 (13.9)	Ref
Yes	182 (2.7)	1.26 (0.91–1.74)	603 (6.8)	1.15 (0.95–1.39)	452 (22.7)	0.94 (0.71–1.24)	913 (14.3)	1.01 (0.86–1.19)
**Externalizing symptoms**								
No	438 (1.5)	Ref	1362 (4.6)	Ref	986 (21.0)	Ref	2049 (13.9)	Ref
Yes	30 (6.0)	**1.95 (1.04–3.65)**	107 (8.7)	0.93 (0.65–1.33)	73 (22.1)	0.93 (0.54–1.58)	187 (16.2)	**1.38 (1.01–1.90)**
**Current use of ENDS**								
No	445 (1.5)	Ref	1261 (4.3)	Ref	853 (20.1)	Ref	1913 (13.6)	Ref
Yes	23 (23.6)	**5.47 (1.75–17.14)**	210 (17.9)	**2.67 (1.96–3.65)**	203 (27.5)	0.91 (0.61–1.34)	326 (17.9)	**1.27 (1.02–1.59)**
**Current other tobacco use**								
No	437 (1.5)	Ref	1261 (4.2)	Ref	847 (21.4)	Ref	1821 (13.5)	Ref
Yes	31 (5.3)	**2.29 (1.04–5.01)**	211 (10.2)	**1.79 (1.34–2.41)**	215 (19.8)	**0.49 (0.33–0.71)**	420 (17.7)	**1.54 (1.25–1.90)**
**Past 12-month cannabis use**^b^								
No	291 (1.2)	Ref	892 (3.6)	Ref	623 (21.5)	Ref	1312 (13.4)	Ref
Yes	85 (6.3)	**2.14 (1.50–3.06)**	229 (7.9)	**1.74 (1.39–2.19)**	151 (20.8)	0.94 (0.68–1.31)	314 (13.3)	0.88 (0.73–1.07)
**Nicotine harm perception**								
None or little harm	111 (3.3)	Ref	437 (8.9)	Ref	410 (22.7)	Ref	768 (12.6)	Ref
Very harmful	357 (1.4)	0.95 (0.58–1.56)	1027 (3.9)	0.75 (0.56–1.01)	645 (20.4)	0.81 (0.58–1.12)	1461 (14.8)	1.22 (0.99–1.51)
**Cigarette absolute harm perception**								
None or little harm	39 (3.7)	Ref	218 (11.3)	Ref	296 (22.0)	Ref	547 (11.0)	Ref
Very harmful	429 (1.5)	0.67 (0.37–1.22)	1245 (4.4)	**0.67 (0.50–0.91)**	763 (21.0)	0.80 (0.58–1.11)	1687 (15.3)	**1.47 (1.18–1.83)**
**Comparative harm perception**								
Less harmful	165 (2.9)	Ref	493 (5.6)	Ref	293 (20.4)	Ref	667 (15.8)	Ref
Equally or more harmful	303 (1.3)	0.75 (0.45–1.24)	969 (4.4)	1.05 (0.85–1.30)	750 (21.4)	0.96 (0.70–1.30)	1545 (13.5)	0.95 (0.79–1.14)
**Cigarette nicotine dependence**^c^	─	─	─	─	29.5 (1.0)^c^	**1.03 (1.02–1.03)**	29.4 (0.8)^c^	**0.98 (0.97–0.98)**

W = wave, Wt% = weighted percentage, CI = confidence interval, LGB = Lesbian, Gay, bisexual, or something else, ENDS = electronic nicotine delivery system, Ref = reference.

All analyses were weighted using PATH Wave 5 all-wave weights.

a Unweighted n and weighted row percentages.

b Missing for cannabis use was >5% within transition outcomes.

c Nicotine dependence shows the weighted mean and standard error among individuals who progressed or discontinued using ENDS.

d No observation was in this category or the model did not converge. Bold-faced indicates *p* < 0.05.

#### Relapse

Of the 15,156 adult former cigarette smokers, a population estimate of 4.8% reported a relapse in smoking over W4–W5 (Fig. 2 and Table 2). Relapse in cigarette smoking was less likely among older age groups than young adults (AOR range = 0.08–0.63), those with some college (AOR = 0.59; 95% CI: 0.46–0.76) or bachelor’s or more (AOR = 2.34; 95% CI: 1.88–2.92) than below high school education, and those who made an annual income of $100,000 or more compared with income of <$25,000 (AOR = 0.53; 95% CI: 0.30–0.93) (Table 4). Relapse was more likely among adults who were current ENDS users (vs. non-current users; AOR = 2.67; 95% CI: 1.96–3.65), current other tobacco products users (vs. non-current users; AOR = 1.79; 95% CI: 1.34–2.41), and past 12-month cannabis user (vs. non-users; AOR = 1.79; 95% CI: 1.39–2.19) (Table 4). Perceiving cigarettes as very harmful to health (vs. none or little harm; AOR = 0.67; 95% CI: 0.50–0.91) was associated with lower odds of relapse in cigarette smoking (Table 4).

#### Progression

Of the 4632 adult periodic smokers, a population estimate of 21.1% reported progression in cigarette smoking over W4–W5 (Fig. 2 and Table 2). Progression in cigarette smoking use was less likely among Hispanic (vs. white, AOR = 0.51; 95% CI: 0.36–0.74). Adults with some college education (vs. less than high school; AOR = 0.63; 95% CI: 0.43–0.92), bachelor’s degree or more (vs. less than high school; AOR = 0.45; 95% CI: 0.27–0.75), and those who were current other tobacco products users (vs. non-current users; AOR = 0.49; 95% CI: 0.33–0.71) were less likely to progress in cigarette smoking. Smokers with a high nicotine dependence rate (AOR = 1.03; 95% CI: 1.02–1.04) were more likely to progress in cigarette smoking than low dependent smokers (Table 4).

#### Cessation

Of the 16,147 adult current experimental or established cigarette smokers, a population estimate of 14.0% reported cessation in smoking over W4–W5 (Fig. 2 and Table 2). Cessation in cigarette smoking was less likely among older age groups than young adults (AOR range = 0.45–0.56). Adults with some college education (vs. less than high school; AOR = 1.28; 95% CI: 1.04–1.57), bachelor’s degree or more (vs. less than high school; AOR = 1.92; 95% CI: 1.47–2.51), and those who made an annual income of $100,000 or more compared with income of <$25,000 (AOR = 1.57; 95% CI: 1.17–2.10) had greater odds of discontinuing cigarette smoking over the follow-up period. Adult smokers with externalizing symptoms (vs. no symptoms, AOR = 1.38; 95% CI: 1.01–1.90), were current ENDS users (vs. non-current users; AOR = 1.27; 95% CI: 1.02–1.5), current other tobacco products users (vs. non-current users; AOR = 1.54; 95% CI: 1.25–1.90) were more likely to discontinue smoking at the follow-up. Perceiving cigarettes as very harmful to health (vs. none or little harm; AOR = 1.47; 95% CI: 1.18–1.83) was associated with cessation of cigarette smoking (Table 4). Smokers with a high nicotine dependence rate were less likely to have stopped smoking by follow-up (AOR = 0.98; 95% CI: 0.97–0.98) (Table 4).

The pattern of findings remained largely unchanged when we included the replenishment sample and performed multivariable regression analysis for paired waves (see Supplementary Tables S4–S7). While the initiation or relapse to cessation ratio of ENDS increased over the W3–W5 (ratio = 1.4), the same ratio for cigarettes decreased (ratio = 0.8), indicating a high number of adults initiating ENDS use, while the number of cigarette smokers decrease over time (Table 2).

Of the 941 adults who were exclusive current established ENDS users at W4, an estimated 63.2% stayed exclusive ENDS users, 6% became exclusive cigarette smokers, 8.2% dual users, and 22.6% became non-users after 1 year (Supplementary Fig. S2). Of the 8845 adults who were exclusive current established cigarette users at W4, an estimated 78.8% stayed exclusive smokers, 2.3% became exclusive cigarette smokers, 6.1% dual users, and 12.8% became non-users after 1 year. Of the 1064 dual users at W4, an estimated 46.9% remained dual users but 32.8% became exclusive cigarette smokers, 13.2% were exclusive ENDS users, and the remaining 7.1% became non-users of either product. A similar pattern was observed for W3–W4 time frame.

## Discussion

Information on the initiation, relapse, progression, and cessation of tobacco product use is important to support successful tobacco control policies and programs. The observed patterns of transitions in ENDS and cigarette use across the three most recent waves of PATH Study suggest a high degree of variability in use status among US adults, which is in line with earlier waves (W1 & W2) of this national survey.^46^ This variability was more pronounced in some priority populations who are disproportionately affected by tobacco products use, including young adults, or people with mental health conditions. Evidence-based efforts and broader policy measures are critical to reducing the burden of tobacco products use on this population via preventing initiation, relapse, and progression and promoting cessation.

Our findings shed some light on trends in vaping and smoking among adults in the US. From 2015/2018 to 2018/2019, the ratio of people who started to those who ceased ENDS use increased substantially, while the same ratio for cigarette smoking decreased. A study from five waves of PATH data that examined the cessation rates among US adults revealed that discontinuing past 30-day ENDS use sharply declined between 2016/2017 and 2018/2019 relative to prior years (2013–2015).^47^ The authors linked this change to the advent of high-tech nicotine salt-based e-liquid formulations, which were available on June 2015, with JUUL products dominating the market since then.^47^ In the same study,^47^ quitting increased for cigarette smokers during the same period, which was consistent with national cross-sectional data.^48^ Current ENDS users at baseline (for each study period) were more likely to have started smoking at follow-up, and the converse was true as well (smoking was associated with starting to vape), in line with prior research.^49^^,^^50^ Similarly, established ENDS users at baseline were more likely to have discontinued smoking at follow-up, and the converse was true. These findings show substantial variability in starting and quitting both products that may complicate cessation interventions, especially when previous reports show that dual users of cigarettes and ENDS were more likely to quit vaping than quit smoking, which thwart the cessation potential of ENDS and public health interventions in general.^51^ This was also observed in our study showing that after 1 year follow-up (W4–W5), while 32.8% of dual users became exclusive smokers, only 13.2% became exclusive ENDS users.

An interesting finding of our study was that people with internalizing mental health symptoms were more prone to initiate ENDS, while those with externalizing symptoms were more prone to initiate cigarettes over the time period. Those who also experienced internalizing symptoms were at greater risk of progression in ENDS use. Previous PATH Study^23^ showed that internalizing and externalizing problems are found to be more prevalent among ENDS and/or cigarettes either exclusive or dual use. Significant comorbidity exists between tobacco product use and mental illnesses, a vulnerability exploited by the tobacco industry that targets these susceptible people.^52^ According to the CDC, 1 in 3 adults with mental illness smoke compared to 1 in 5 without this condition.^53^ Tobacco smoking is the largest single reason why people with mental health conditions have a life expectancy 10–20 years lower than the general population.^54^ Although vaping-induced mental health complications are yet to be fully explored, growing evidence^55^, ^56^, ^57^ suggests that vaping may be associated with mental health conditions similar to those observed in other tobacco product users, especially cigarette smokers. It is crucial to consider mental health burdens as regulatory implications for novel tobacco products, including ENDS.^57^ As alluded to above, evidence- and population-based prevention strategies and cessation treatments must be protracted to people with internalizing and externalizing mental health symptoms, who are venerable to initiate and sustain using tobacco products.

In our study, higher perceived nicotine harm was associated with discontinuing vaping but not cigarettes use. Higher nicotine perception that leads to cessation in ENDS use among “exclusive” users would benefit both users and public health in general. However, some smokers, with no plans to quit, use ENDS to wean off cigarettes discontinuing both vaping and cigarettes as a final endpoint. If higher nicotine perceived harm leads to quitting ENDS but not cigarettes among dual users, this may undermine the potential competence of ENDS use to aid smokers to quit. Future studies are needed to explore how nicotine perceived harm in relation to specific diseases like cancer can lead to intended consequences (quit cigarettes and use ENDS, or quit both cigarettes and ENDS) and unintended consequences (quit ENDS but not cigarettes) among dual users of these products over time. Our findings also showed higher absolute and comparative harm perception were effective for both products. For example, those who believed ENDS is very harmful (vs. less harmful) were less likely to start and more likely to quit ENDS. This was the same for cigarette smokers. It is, therefore, important to continue raising awareness and accurate risk communication about the potential effects of novel products like ENDS and the well-established health consequences of smoking to increase harm perceptions, while avoiding unintended consequences (e.g., deter adopting ENDS or driving users back to smoking).^58^

In our study, past 12-month cannabis use was associated with initiation and relapse for both ENDS and cigarette use. While the gateway hypothesis suggests that tobacco use leads to cannabis use,^59^ our findings showed that cannabis use also can lead to initiation and relapse of cigarette smoking^60^ and also ENDS use. The findings from this study provide evidence that is needed to develop innovative tobacco control policies that integrate cannabis-related prevention approaches into existing tobacco control strategies. Future longitudinal studies are needed to better understand whether initiation of ENDS is more strongly associated with cannabis smoking/vaping (or vice versa) and whether the specific characteristics of youth and adults who constitute each pattern is instrumental to optimize the prevention interventions to protect public health.

Our findings have some limitations. First, our results do not account for participants’ longer-term ENDS or cigarette use histories, such as previous quit attempts, which may affect future transitions. Nevertheless, this study covers a 4-year span for a well-defined and nationally representative cohort. Second, our study did not account for some aspects that are likely to influence behavioral transitions in ENDS and cigarette use, including intensity of use, nicotine concentration, ENDS device characteristics, and information about participants’ behaviors between study interviews.^46^ Third, patterns of use may be continuing to evolve, so the generalizability of the findings to current use patterns remains to be established. Fourth, this study did not include adolescents since they have distinct tobacco product use patterns compared to adults. Future similar studies, including additional waves of the PATH Study, are warranted to explore the same longitudinal transitions in the adolescent population. Fifth, the study design supports limited but not strong causal inferences. Last, the studied variables were self-reported and might be subject to recall and response bias. Nonetheless, PATH Study questions are developed using a careful protocol and provide reliable and valid information about tobacco use behavior.^61^

In summary, the findings of this nationally representative study add to the growing literature on the behavioral transitions in ENDS and cigarette use among US adults. Our results showed that discontinuing ENDS use was common among US adults. However, in absolute terms, ENDS use was growing, while smoking was falling. The current established ENDS use did not discourage relapse to cigarette smoking among former smokers, nor smoking initiation among never smokers. Conversely, current established cigarette smoking encouraged relapse to ENDS use among former users and initiation to vape among never ENDS users. Although it is difficult to conclude that increasing trends in ENDS use predict decreasing trends in cigarette use, ENDS may play a role in this decline, which needs to be explored in future longitudinal studies. Tobacco control programs should account for behavioral transitions focusing on the priority population, including young adults and tobacco products users who co-use other substances (e.g., cannabis). They also must focus on internalizing and externalizing mental health symptoms that were observed differently for ENDS and cigarettes transitions in this study.

## Contributors

MEK: conceptualization, data curation, formal analysis, methodology, supervision, validation, visualisation, writing—original draft, and writing—review & editing.

NTB: conceptualization, funding acquisition, methodology, resources, validation, writing—original draft, and writing—review & editing.

MEK and NTB directly accessed and verified the underlying data reported in the manuscript and are responsible for the decision to submit the manuscript.

## Data sharing statement

The Population Assessment of Tobacco and Health (PATH) data underlying this article are available from the Interuniversity Consortium for Political and Social Research at https://dx.doi.org/10.3886/SERIES606. Our code is available upon request to the corresponding author.

## Declaration of interests

We declare no competing interests.
