# Supplementary material for: Longitudinal transitions in e-cigarette and cigarette use among US adults: prospective cohort study
Source: Lancet Reg Health Am. 2023 May 16;22:100508. doi: 10.1016/j.lana.2023.100508 (PMC10205448; doi:10.1016/j.lana.2023.100508)
Supplement: Supplementary Figs. S1 and S2 and Supplementary Tables S1–S7 [file mmc1.pdf]

Supplementary materials

Study title: Longitudinal transitions in e-cigarette and cigarette use among US adults: Prospective cohort study
Authors: Mohammad Ebrahimi Kalan, Ph.D., Noel T. Brewer, Ph.D.

Table of Contents

Weighted data presentation 2

Abbreviations 2

Supplementary Table 1. Tobacco products use definitions, PATH Study 3

Supplementary Table 2. Definitions of behavioral transitions in ENDS or cigarettes use, PATH Study 4

Supplementary Table-3. Items for internalizing and externalizing symptoms, PATH Study 5

Supplementary Figure 1. Study Flow Diagram 6

Supplementary Table 4. Multivariable analysis of ENDS or cigarettes use initiation among adults across W3-W4 and W4-W5, PATH Study 7

Supplementary Table 5. Multivariable analysis of relapse in ENDS or cigarette use among adults across W3-W4 and W4-W5, PATH Study 8

Supplementary Table 6. Multivariable analysis of progression in ENDS and cigarettes use among adults across W3-W4 and W4-W5, PATH Study 9

Supplementary Table 7. Multivariable analysis of ENDS or cigarettes use cessation among adults across W3-W4 and W4-W5, PATH Study 10

Supplementary Figure 2. Between products (ENDS to cigarettes or vice versa) transitions from W3-W4 (above) and W4-W5 (bottom), PATH Study 11

Weighted data presentation

All percentages presented in this supplementary are weighted using SAS procedures to provide nationally representative estimates of US adults and unbiased measures, adjusting for differences in selection probability and nonresponse while accounting for design features.

Abbreviations

PATH=Population Assessment on Tobacco and Health
ENDS=Electronic nicotine delivery system
W=Wave
Wt%=Weighted percentages
AOR= Adjusted odds ratio
CI=Confidence interval,
Ref=Reference
LGB= Lesbian, Gay, Bisexual, or Something else

Supplementary Table 1. Tobacco products use definitions, PATH Study

Product	Use status	Definitions
**ENDS**	Never use	Never used ENDS, even once.
	Ever use	Have ever used any ENDS
	Former established use	Have ever used any ENDS products fairly regularly and have not used them within the past 12 months or currently do not use at all.
	Former experimental use	Have never used any ENDS fairly regularly, have not used them within the past 12 months or currently use not at all.
	Current established use	Have ever used any ENDS, have ever used them fairly regularly, and currently use every day or some days.
	Current experimental use	Have never used any ENDS fairly regularly, and currently use every day or some days.
	Daily use	Have ever used any ENDS, have ever used them fairly regularly, and currently use every day.
	Some days use	Have ever used any ENDS, have ever used them fairly regularly, and currently use some days.
**Cigarettes**	Never use	Never used cigarettes, even once.
	Ever use	Have ever smoked a cigarette, used <100 cigarettes in lifetime
	Former established use	Have smoked at least 100 cigarettes in their lifetime, and have not smoked them within the past 12 months or currently smoke not at all.
	Former experimental use	Have not smoked at least 100 cigarettes in their lifetime, and did not smoke them within the past 12 months or currently smoke not at all.
	Current established use	Have smoked at least 100 cigarettes in their lifetime, and currently smoke every day or some days
	Current experimental use	Have not smoked at least 100 cigarettes in their lifetime, and currently smoke every day or some days
	Daily use	Have smoked at least 100 cigarettes in their lifetime, and currently smoke every day
	Some days use	Have smoked at least 100 cigarettes in their lifetime, and currently smoke some days

Supplementary Table 2. Definitions of behavioral transitions in ENDS or cigarettes use, PATH Study

Behavioral transitions	Definitions	Coded
**Initiation**	Transition from never use to ever use of [ENDS/cigarettes] across timepoints.	0=no or 1=yes
**Relapse**	Transition from former established or experimental use of [ENDS/cigarettes] to current established or experimental use of [ENDS/cigarettes] across timepoints.	0=no or 1=yes
**Progression**	Transition from some days established use to everyday established use, or from current experimental use to some days or everyday established use across timepoints.	0=no or 1=yes
**Cessation**	Transition from current established or current experimental use of [ENDS/cigarettes] to former use across timepoints.	0=no or 1=yes

Supplementary Table-3. Items for internalizing and externalizing symptoms, PATH Study

**Internalizing Symptoms***
When was the last time that you had significant problems with ...
1) Feeling very trapped, lonely, sad, blue, depressed, or hopeless about the future?
2) Sleep trouble, such as bad dreams, sleeping restlessly, or falling asleep during the day?
3) Feeling very anxious, nervous, tense, scared, panicked, or like something bad was going to happen?
4) Becoming very distressed and upset when something reminded you of the past?
**Externalizing Symptoms**
When was the last time that you did any of the following things two or more
1) Lied or conned to get things you wanted or to avoid having to do something?
2) Had a hard time paying attention at school, work, or home?
3) Had a hard time listening to instructions at school, work, or home?
4) Were a bully or threatened other people?
5) Started physical fights with other people?
6) Felt restless or the need to run around or climb on things?
7) Gave answers before the other person finished asking the question?

*Response options for all questions were: "0" (never), "1" (over a year ago), "2" (2 to 12 months). Participants were grouped into two categories: those who never reported any problems (coded as 0) and those who reported at least one problem in the past 12 months or more (coded as 1).

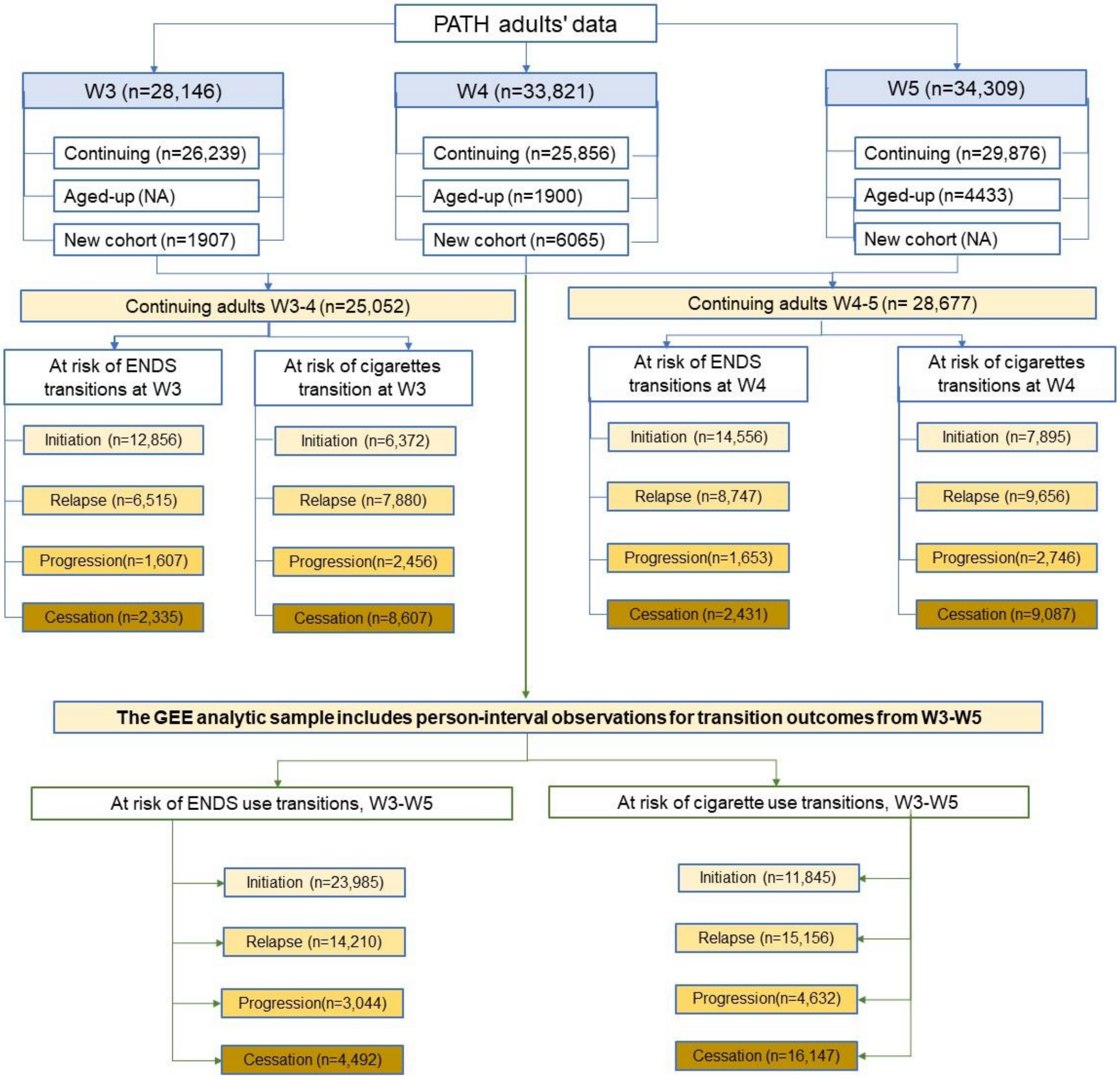

Supplementary Figure 1. Study Flow Diagram

Supplementary Table 4. Multivariable analysis of ENDS or cigarettes use initiation among adults across W3-W4 and W4-W5, PATH Study

Characteristics	Initiation of ENDS use		Initiation of cigarette use	
	**Waves 3-4 n=12,856**	**Waves 4-5 n=14,556**	**Waves 3-4 n=6,372**	**Waves 4-5 N=7,895**
	aOR (95% CI)	aOR (95% CI)	aOR (95% CI)	aOR (95% CI)
**Age group**				
18–24	Ref	Ref	Ref	Ref
25–44	**0.26 (0.16-0.41)**	**0.19 (0.14-0.25)**	0.17 (0.09-0.31)	0.14 (0.08-0.25)
45–64	**0.17 (0.10-0.28)**	**0.09 (0.06-0.14)**	¥	¥
65+	**0.03 (0.01-0.07)**	**0.04 (0.02-0.07)**	¥	¥
**Sex**				
**Female**	Ref	Ref	Ref	Ref
**Male**	1.07 (0.74-1.55)	1.26 (0.97-1.64)	**2.12 (1.17-3.85)**	**1.54 (1.03-2.31)**
**Sexual Orientation**				
Straight	Ref	Ref	Ref	Ref
LGB	1.23 (0.81-1.88)	1.28 (0.70-2.35)	**0.48 (0.25-0.90)**	1.84 (0.98-3.46)
Not reported	¥	0.21 (0.01-3.03)	1.31 (0.17-10.15)	4.17 (0.98-17.82)
**Race/ethnicity**				
Non-Hispanic White	Ref	Ref	Ref	Ref
Non-Hispanic Black	1.11 (0.70-1.77)	1.19 (0.80-1.78)	1.32 (0.64-2.69)	0.81 (0.46-1.42)
Hispanic	1.31 (0.82-2.08)	**1.38 (1.00-1.90)**	1.57 (0.90-2.73)	1.43 (0.82-2.49)
Other races	0.77 (0.36-1.61)	0.71 (0.48-1.06)	1.69 (0.44-6.56)	1.66 (0.70-3.94)
**Educational**				
Less than high school	Ref	Ref	Ref	Ref
High school graduate	0.88 (0.53-1.46)	0.85 (0.61-1.19)	0.76 (0.39-1.50)	0.51 (0.25-1.03)
Some college	0.88 (0.55-1.40)	0.99 (0.70-1.40)	0.66 (0.32-1.35)	0.56 (0.29-1.11)
Bachelor’s degree or more	0.57 (0.30-1.06)	**0.62 (0.41-0.94)**	0.61 (0.20-1.84)	**0.29 (0.09-0.97)**
**Income**				
Less than $25,000	Ref	Ref	Ref	Ref
$25,000 to $49,999	0.91 (0.55-1.49)	1.37 (0.94-1.99)	**0.45 (0.20-0.99)**	1.06 (0.57-1.98)
$50,000 to $99,999	0.97 (0.56-1.70)	1.13 (0.77-1.66)	0.52 (0.25-1.08)	**0.39 (0.22-0.73)**
$100,000 or more	0.96 (0.54-1.71)	1.54 (0.96-2.47)	1.21 (0.58-2.52)	0.69 (0.37-1.28)
Not reported	0.90 (0.44-1.86)	1.23 (0.64-2.35)	0.97 (0.43-2.16)	0.51 (0.22-1.19)
**Internalizing symptoms**				
No	Ref	Ref	Ref	Ref
Yes	**1.77 (1.18-2.64)**	**1.72 (1.33-2.22)**	1.32 (0.79-2.23)	**1.61 (1.01-2.56)**
**Externalizing symptoms**				
No	Ref	Ref	Ref	Ref
Yes	1.00 (0.51-1.97)	1.28 (0.73-2.24)	0.50 (0.16-1.58)	1.07 (0.67-1.73)
**Current use of cigarettes/ENDS** [a]				
No	Ref	Ref	Ref	Ref
Yes	**5.56 (3.70-8.37)**	**5.13 (3.39-7.78)**	2.97 (0.91-9.63)	**6.83 (1.04-44.78)**
**Current other tobacco use**				
No	Ref	Ref	Ref	Ref
Yes	**2.88 (1.73-4.81)**	**2.08 (1.27-3.41)**	1.80 (0.52-6.16)	**2.74 (1.08-6.99)**
**Past 12-month cannabis use**				
No	Ref	Ref	Ref	
Yes	**2.17 (1.36-3.46)**	**3.49 (2.47-4.93)**	**2.14 (1.21-3.78)**	**2.17 (1.50-3.13)**
**Nicotine harm perception**				
None or little harm	Ref	Ref	Ref	Ref
Very harmful	1.07 (0.73-1.56)	0.73 (0.51-1.04)	0.66 (0.28-1.54)	1.14 (0.69-1.88)
**ENDS/Cigarette absolute harm perception** [b]				
None or little harm	Ref	Ref	Ref	Ref
Very harmful	**0.64 (0.43-0.96)**	0.94 (0.69-1.27)	0.72 (0.27-1.95)	0.77 (0.37-1.59)
**Comparative harm perception**				
Less harmful	Ref	Ref	Ref	Ref
Equally or more harmful	0.85 (0.58-1.26)	1.03 (0.76-1.39)	0.63 (0.29-1.40)	0.87 (0.56-1.35)

Note. Regression models were weighted using wave 4 all-wave weights (for Waves 3-4) and wave 5 all-wave weights. (for Waves 4-5). Bold-faced aOR indicates p<.05.

[a] First two columns show current established cigarettes smoking and the last two columns show current established ENDS use.

[b] First two columns indicate ENDS absolute harm perception, and the last two columns indicate cigarettes' absolute harm perception. ¥ No observation was in this category or the model did not converge.

Supplementary Table 5. Multivariable analysis of relapse in ENDS or cigarette use among adults across W3-W4 and W4-W5, PATH Study

Characteristics	Relapse of ENDS use		Relapse of cigarette use	
	**Waves 3-4 n=6,515**	**Waves 4-5 n=8,747**	**Waves 3-4 n=7,880**	**Waves 4-5 N=7,895**
	aOR (95% CI)	aOR (95% CI)	aOR (95% CI)	aOR (95% CI)
**Age group**				
18–24	Ref	Ref	Ref	Ref
25–44	**0.70 (0.54-0.92)**	**0.45 (0.35-0.59)**	**0.62 (0.47-0.82)**	**0.59 (0.44-0.79)**
45–64	**0.48 (0.34-0.68)**	**0.28 (0.19-0.42)**	**0.26 (0.18-0.37)**	**0.27 (0.18-0.39)**
65+	0.62 (0.31-1.23)	**0.27 (0.12-0.60)**	**0.09 (0.06-0.16)**	**0.06 (0.03-0.11)**
**Sex**				
Female	Ref	Ref	Ref	Ref
Male	0.89 (0.68-1.17)	0.93 (0.74-1.17)	0.90 (0.71-1.15)	0.91 (0.71-1.15)
**Sexual Orientation**				
Straight	Ref	Ref	Ref	Ref
LGB	1.51 (0.99-2.29)	0.99 (0.71-1.37)	1.28 (0.89-1.86)	1.04 (0.69-1.59)
Not reported	2.14 (0.71-6.47)	1.37 (0.46-4.07)	0.84 (0.15-4.72)	1.78 (0.28-11.10)
**Race/ethnicity**				
Non-Hispanic White	Ref	Ref	Ref	Ref
Non-Hispanic Black	1.49 (0.96-2.32)	0.75 (0.47-1.20)	1.26 (0.79-2.02)	1.23 (0.86-1.77)
Hispanic	1.55 (1.09-2.22)	0.80 (0.59-1.08)	1.07 (0.69-1.66)	0.89 (0.64-1.24)
Other races	1.45 (0.90-2.34)	1.10 (0.77-1.57)	0.80 (0.52-1.22)	1.18 (0.66-2.11)
**Educational**				
Less than high school	Ref	Ref	Ref	Ref
High school graduate	1.10 (0.76-1.61)	1.02 (0.73-1.44)	**0.63 (0.42-0.95)**	0.77 (0.54-1.11)
Some college	1.00 (0.68-1.47)	1.21 (0.90-1.63)	**0.48 (0.35-0.65)**	**0.66 (0.44-0.97)**
Bachelor's degree or more	1.09 (0.68-1.74)	0.93 (0.61-1.43)	**0.42 (0.27-0.64)**	**0.47 (0.29-0.75)**
**Income**				
Less than $25,000	Ref	Ref	Ref	Ref
$25,000 to $49,999	1.26 (0.89-1.77)	0.89 (0.67-1.19)	0.87 (0.66-1.16)	**0.52 (0.37-0.73)**
$50,000 to $99,999	1.05 (0.71-1.54)	0.86 (0.61-1.21)	**0.66 (0.46-0.96)**	**0.45 (0.32-0.64)**
$100,000 or more	1.18 (0.78-1.80)	0.89 (0.61-1.30)	**0.42 (0.28-0.64)**	**0.30 (0.19-0.48)**
Not reported	0.95 (0.50-1.79)	0.67 (0.41-1.11)	0.76 (0.37-1.54)	0.55 (0.27-1.15)
**Internalizing symptoms**				
No	Ref	Ref	Ref	Ref
Yes	**1.33 (1.04-1.70)**	1.28 (0.99-1.67)	1.08 (0.83-1.39)	1.23 (0.96-1.58)
**Externalizing symptoms**				
No	Ref	Ref	Ref	Ref
Yes	1.05 (0.66-1.67)	**1.40 (1.00-1.94)**	0.86 (0.50-1.46)	1.09 (0.68-1.76)
**Current use of cigarettes/ENDS** [a]				
No	Ref	Ref	Ref	Ref
Yes	**2.47 (1.82-3.36)**	**2.25 (1.71-2.97)**	**3.52 (2.14-5.77)**	**2.36 (1.51-3.68)**
**Current other tobacco use**				
No	Ref	Ref	Ref	Ref
Yes	**1.51 (1.02-2.24)**	**1.43 (1.10-1.86)**	1.79 (1.18-2.74)	**1.72 (1.15-2.57)**
**Past 12-month cannabis use**				
No	Ref	Ref	Ref	
Yes	1.28 (0.89-1.84)	1.23 (0.96-1.59)	**1.81 (1.36-2.41)**	**1.67 (1.18-2.38)**
**Nicotine harm perception**				
None or little harm	Ref	Ref	Ref	Ref
Very harmful	0.84 (0.65-1.09)	1.11 (0.85-1.46)	0.91 (0.60-1.39)	**0.70 (0.51-0.94)**
**ENDS/Cigarette absolute harm perception** [b]				
None or little harm	Ref	Ref	Ref	Ref
Very harmful	**0.64 (0.47-0.87)**	0.81 (0.63-1.05)	**0.56 (0.34-0.91)**	0.67 (0.45-1.00)
**Comparative harm perception**				
Less harmful	Ref	Ref	Ref	Ref
Equally or more harmful	0.90 (0.71-1.14)	**0.72 (0.56-0.93)**	**1.81 (1.36-2.41)**	1.22 (0.89-1.66)

Note. Regression models were weighted using wave 4 all-wave weights (for Waves 3-4) and wave 5 all-wave weights. (for Waves 4-5). Bold-faced aOR indicates p<.05.

[a] First two columns show current established cigarettes smoking and the last two columns show current established ENDS use.

[b] First two columns indicate ENDS absolute harm perception, and the last two columns indicate cigarettes' absolute harm perception.

Supplementary Table 6. Multivariable analysis of progression in ENDS and cigarettes use among adults across W3-W4 and W4-W5, PATH Study

Characteristics	Progression in ENDS use		Progression in cigarettes use	
	**Waves 3-4 n=1,611**	**Waves 4-5 n=1,653**	**Waves 3-4 n=2,467**	**Waves 4-5 N=2,746**
	aOR (95% CI)	aOR (95% CI)	aOR (95% CI)	aOR (95% CI)
**Age group**				
18–24	Ref	Ref	Ref	Ref
25–44	1.85 (0.80-4.27)	0.37 (0.11-1.21)	**0.62 (0.39-0.99)**	1.14 (0.73-1.79)
45–64	0.70 (0.26-1.93)	**0.34 (0.13-0.87)**	**0.52 (0.27-0.99)**	1.17 (0.70-1.97)
65+	0.49 (0.06-3.82)	0.19 (0.01-2.63)	**0.32 (0.13-0.80)**	0.88 (0.31-2.48)
**Sex**				
Female	Ref	Ref	Ref	Ref
Male	1.82 (0.82-4.05)	083 (0.29-2.37)	0.99 (0.64-1.54)	0.84 (0.54-1.30)
**Sexual Orientation**				
Straight	Ref	Ref	Ref	Ref
LGB	1.02 (0.27-3.77)	1.04 (0.33-3.31)	1.29 (0.68-2.45)	1.35 (0.74-2.46)
Not reported	¥	2.12 (0.46-9.90)	1.54 (0.40-5.97)	0.22 (0.01-7.57)
**Race/ethnicity**				
Non-Hispanic White	Ref	Ref	Ref	Ref
Non-Hispanic Black	0.36 (0.06-2.16)	2.12 (0.46-9.90)	**0.28 (0.12-0.66)**	1.14 (0.62-2.09)
Hispanic	**0.14 (0.04-0.48)**	0.67 (0.16-2.76)	**0.44 (0.25-0.76)**	0.61 (0.36-1.04)
Other races	0.54 (0.14-2.09)	1.34 (0.20-8.94)	1.02 (0.44-2.37)	0.57 (0.28-1.18)
**Educational**				
Less than high school	Ref	Ref	Ref	Ref
High school graduate	2.18 (0.52-9.14)	0.94 (0.32-2.72)	0.76 (0.44-1.32)	0.76 (0.42-1.38)
Some college	1.83 (0.53-6.28)	1.37 (0.51-3.68)	0.76 (0.44-1.34)	0.62 (0.35-1.09)
Bachelor's degree or more	3.43 (0.77-15.19)	3.11 (0.71-13.65)	0.61 (0.31-1.21)	**0.37 (0.18-0.75)**
**Income**				
Less than $25,000	Ref	Ref	Ref	Ref
$25,000 to $49,999	0.34 (0.11-1.03)	0.58 (0.15-2.22)	0.74 (0.43-1.26)	0.85 (0.53-1.38)
$50,000 to $99,999	0.73 (0.28-1.91)	0.50 (0.14-1.77)	0.77 (0.40-1.46)	0.58 (0.34-1.01)
$100,000 or more	0.45 (0.13-1.59)	1.62 (0.40-6.46)	0.50 (0.21-1.16)	0.76 (0.40-1.47)
Not reported	0.68 (0.14-3.36)	0.42 (0.06-3.02)	0.71 (0.21-2.46)	1.22 (0.58-2.58)
**Internalizing symptoms**				
No	Ref	Ref	Ref	Ref
Yes	1.99 (0.92-4.31)	0.53 (0.25-1.14)	**0.64 (0.40-0.99)**	1.19 (0.83-1.69)
**Externalizing symptoms**				
No	Ref	Ref	Ref	Ref
Yes	0.41 (0.08-2.10)	1.16 (0.26-5.20)	0.69 (0.22-2.16)	1.01 (0.51-2.02)
**Current use of cigarettes/ENDS [a]**				
No	Ref	Ref	Ref	Ref
Yes	**0.35 (0.17-0.72)**	0.93 (0.32-2.73)	0.55 (0.28-1.09)	1.10 (0.63-1.92)
**Current other tobacco use**				
No	Ref	Ref	Ref	Ref
Yes	0.97 (0.39-2.42)	0.25 (0.06-1.00)	0.62 (0.34-1.13)	**0.41 (0.24-0.69)**
**Past 12-month cannabis use**				
No	Ref	Ref	Ref	
Yes	0.87 (0.32-2.40)	1.12 (0.39-3.20)	0.72 (0.43-1.21)	1.13 (0.67-1.89)
**Nicotine harm perception**				
None or little harm	Ref	Ref	Ref	Ref
Very harmful	1.09 (0.53-2.23)	0.80 (0.38-1.71)	1.07 (0.62-1.85)	0.67 (0.41-1.08)
**ENDS/Cigarette absolute harm perception [b]**				
None or little harm	Ref	Ref	Ref	Ref
Very harmful	0.27 (0.06-1.23)	1.10 (0.31-3.94)	1.06 (0.66-1.72)	0.69 (0.47-1.03)
**Comparative harm perception**				
Less harmful	Ref	Ref	Ref	Ref
Equally or more harmful	0.71 (0.27-1.89)	0.78 (0.36-1.71)	0.93 (0.62-1.41)	1.08 (0.64-1.82)
ENDS/cigarette nicotine dependence	**1.03 (1.01-1.05)**	**1.06 (1.03-1.09)**	**1.04 (1.03-1.05)**	**1.02 (1.01-1.03)**

Note. Regression models were weighted using wave 4 all-wave weights (for Waves 3-4) and wave 5 all-wave weights. (for Waves 4-5). Bold-faced aOR indicates $p<.05$.
[a] First two columns show current established cigarettes smoking and the last two columns show current established ENDS use.
[b] First two columns indicate ENDS absolute harm perception, and the last two columns indicate cigarettes' absolute harm perception. [¥] No observation was in this category or model did not converge.

Supplementary Table 7. Multivariable analysis of ENDS or cigarettes use cessation among adults across W3-W4 and W4-W5, PATH Study

**Characteristics**	**Cessation in ENDS use**		**Cessation in cigarette use**	
	**Waves 3-4 n=2,335**	**Waves 4-5 n=2,431**	**Waves 3-4 n=8,607**	**Waves 4-5 N=9,087**
	aOR (95% CI)	aOR (95% CI)	aOR (95% CI)	aOR (95% CI)
**Age group**				
18–24	Ref	Ref	Ref	Ref
25–44	0.83 (0.54-1.27)	0.99 (0.58-1.69)	**0.60 (0.43-0.85)**	0.54 (0.41-0.71)
45–64	0.79 (0.47-1.35)	0.95 (0.53-1.69)	**0.52 (0.35-0.77)**	0.41 (0.30-0.55)
65+	0.85 (0.40-1.83)	1.46 (0.64-3.31)	0.74 (0.41-1.31)	0.93 (0.58-1.48)
**Sex**				
Female	Ref	Ref	Ref	Ref
Male	0.99 (0.69-1.44)	0.98 (0.68-1.42)	0.95 (0.73-1.23)	1.07 (0.86-1.34)
**Sexual Orientation**				
Straight	Ref	Ref	Ref	Ref
LGB	1.17 (0.68-2.01)	0.67 (0.38-1.17)	0.99 (0.58-1.67)	**0.67 (0.47-0.95)**
Not reported	1.90 (0.15-24.49)	¥	0.95 (0.28-3.21)	1.10 (0.27-4.40)
**Race/ethnicity**				
Non-Hispanic White	Ref	Ref	Ref	Ref
Non-Hispanic Black	1.08 (0.52-2.24)	0.59 (0.31-1.10)	0.91 (0.64-1.28)	**0.57 (0.40-0.81)**
Hispanic	1.41 (0.83-2.41)	1.21 (0.65-2.24)	1.08 (0.72-1.62)	1.05 (0.74-1.47)
Other races	1.08 (0.53-2.23)	0.73 (0.38-1.39)	0.63 (0.40-0.98)	1.28 (0.87-1.90)
**Educational**				
Less than high school	Ref	Ref	Ref	Ref
High school graduate	0.92 (0.57-1.49)	1.10 (0.63-1.92)	1.04 (0.66-1.65)	0.89 (0.62-1.27)
Some college	0.97 (0.65-1.46)	1.48 (0.91-2.43)	1.30 (0.91-1.87)	1.25 (0.89-1.75)
Bachelor’s degree or more	0.71 (0.37-1.38)	1.34 (0.65-2.74)	**2.48 (1.57-3.91)**	**1.67 (1.12-2.48)**
**Income**				
Less than $25,000	Ref	Ref	Ref	Ref
$25,000 to $49,999	0.92 (0.59-1.42)	0.85 (0.50-1.45)	0.90 (0.69-1.18)	1.08 (0.80-1.45)
$50,000 to $99,999	0.68 (0.46-1.01)	0.88 (0.51-1.51)	1.11 (0.80-1.55)	1.04 (0.77-1.41)
$100,000 or more	**0.50 (0.29-0.88)**	0.83 (0.44-1.56)	1.48 (0.98-2.26)	**1.56 (1.06-2.31)**
Not reported	0.62 (0.26-1.47)	0.69 (0.27-1.76)	0.95 (0.48-1.90)	1.36 (0.86-2.14)
**Internalizing symptoms**				
No	Ref	Ref	Ref	Ref
Yes	0.87 (0.56-1.36)	1.37 (0.93-2.02)	1.09 (0.83-1.43)	0.91 (0.73-1.15)
**Externalizing symptoms**				
No	Ref	Ref	Ref	Ref
Yes	2.39 (0.99-5.74)	1.19 (0.61-2.33)	1.46 (0.87-2.46)	1.39 (0.95-2.03)
**Current use of cigarettes/ENDS** [a]				
No	Ref	Ref	Ref	Ref
Yes	**1.76 (1.22-2.55)**	**1.54 (1.01-2.34)**	1.35 (0.94-1.95)	1.25 (0.91-1.73)
**Current other tobacco use**				
No	Ref	Ref	Ref	Ref
Yes	1.06 (0.66-1.69)	1.24 (0.78-1.99)	**1.45 (1.07-1.97)**	**1.56 (1.18-2.07)**
**Past 12-month cannabis use**				
No	Ref	Ref	Ref	
Yes	0.92 (0.57-1.49)	0.73 (0.49-1.07)	0.97 (0.65-1.44)	0.89 (0.68-1.16)
**Nicotine harm perception**				
None or little harm	Ref	Ref	Ref	Ref
Very harmful	1.12 (0.73-1.70)	**1.72 (1.23-2.40)**	1.02 (0.74-1.39)	**1.35 (1.01-1.80)**
**ENDS/Cigarette absolute harm perception** [b]				
None or little harm	Ref	Ref	Ref	Ref
Very harmful	**2.07 (1.11-3.86)**	0.94 (0.49-1.81)	1.31 (0.90-1.92)	**1.58 (1.15-2.16)**
**Comparative harm perception**				
Less harmful	Ref	Ref	Ref	Ref
Equally or more harmful	1.42 (0.95-2.12)	1.16 (0.79-1.68)	1.00 (0.80-1.26)	0.89 (0.69-1.13)
ENDS/cigarette nicotine dependence	**0.97 (0.96-0.98)**	**0.98 (0.7-0.99)**	**0.98 (0.97-0.98)**	**0.98 (0.98-0.99)**

Note. Regression models were weighted using wave 4 all-wave weights (for Waves 3-4) and wave 5 all-wave weights. (for Waves 4-5). Bold-faced aOR indicates p<.05.

[a] First two columns show current established cigarettes smoking and the last two columns show current established ENDS use.

[b] First two columns indicate ENDS absolute harm perception, and the last two columns indicate cigarettes' absolute harm perception. ¥ No observation was in this category or model did not converge.

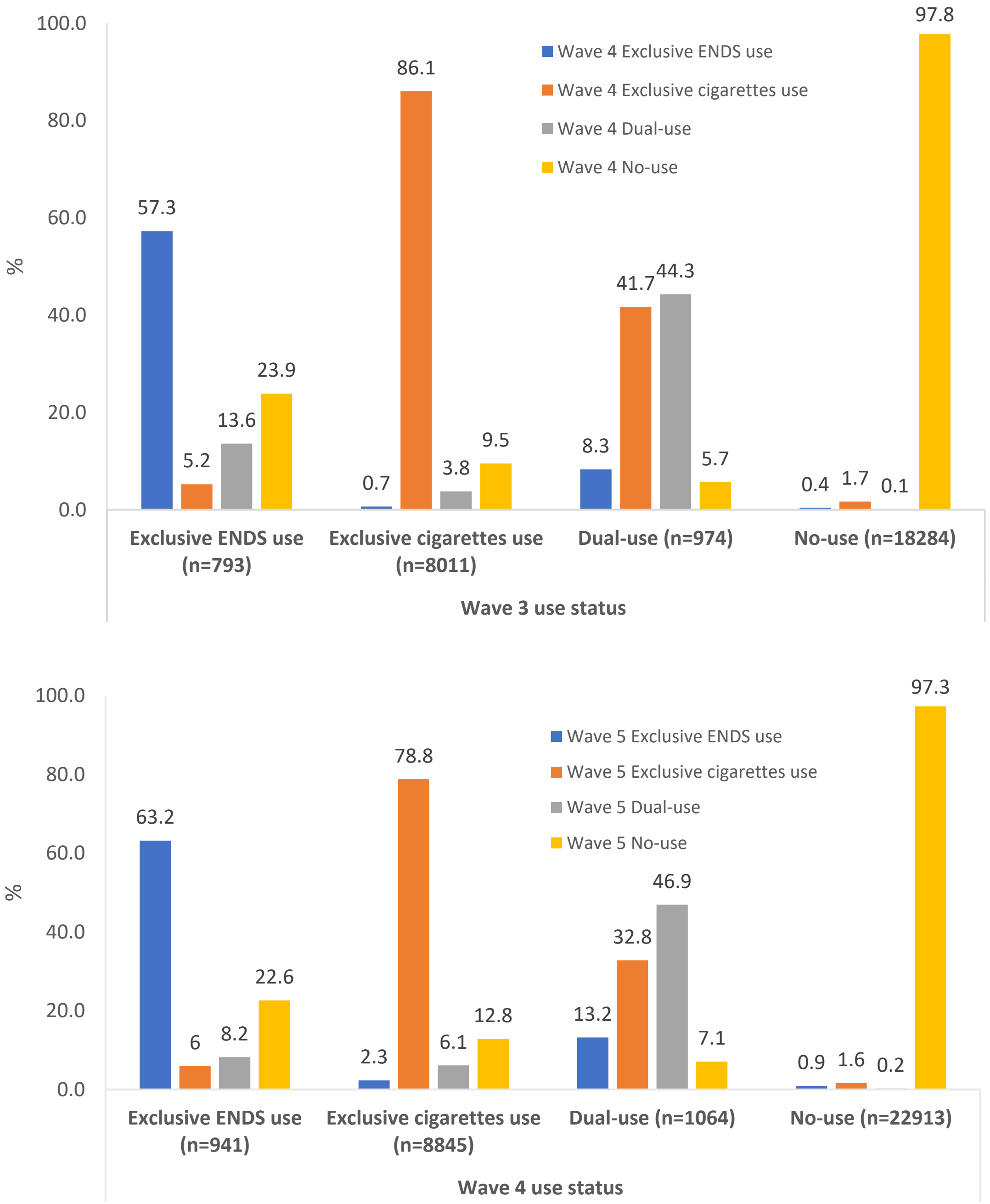

Supplementary Figure 2. Between products (ENDS to cigarettes or vice versa) transitions from W3-W4 (above) and W4-W5 (bottom), PATH Study
Note. These transitions illustrate only the current established use as well as the no-use of each product across waves.
